# PDLIM2 restricts Th1 and Th17 differentiation and prevents autoimmune disease

**DOI:** 10.1186/2045-3701-2-23

**Published:** 2012-06-25

**Authors:** Zhaoxia Qu, Jing Fu, Huihui Ma, Jingjiao Zhou, Meihua Jin, Markus Y Mapara, Michael J Grusby, Gutian Xiao

**Affiliations:** 1 University of Pittsburgh Cancer Institute, Pittsburgh, PA, USA; 2 Department of Microbiology and Molecular Genetics, Pittsburgh, PA, USA; 3 Department of Medicine, University of Pittsburgh School of Medicine, Pittsburgh, PA, USA; 4 Department of Immunology and Infectious Diseases, Harvard School of Public Health, Boston, MA, USA

## Abstract

**Background:**

PDLIM2 is essential for the termination of the inflammatory transcription factors NF-κB and STAT but is dispensable for the development of immune cells and immune tissues/organs. Currently, it remains unknown whether and how PDLIM2 is involved in physiologic and pathogenic processes.

**Results:**

Here we report that naive PDLIM2 deficient CD4^+^ T cells were prone to differentiate into Th1 and Th17 cells. PDLIM2 deficiency, however, had no obvious effect on lineage commitment towards Th2 or Treg cells. Notably, PDLIM2 deficient mice exhibited increased susceptibility to experimental autoimmune encephalitis (EAE), a Th1 and/or Th17 cell-mediated inflammatory disease model of multiple sclerosis (MS). Mechanistic studies further indicate that PDLIM2 was required for restricting expression of Th1 and Th17 cytokines, which was in accordance with the role of PDLIM2 in the termination of NF-κB and STAT activation.

**Conclusion:**

These findings suggest that PDLIM2 is a key modulator of T-cell-mediated immune responses that may be targeted for the therapy of human autoimmune diseases.

## Background

CD4^+^ T helper (Th) cells play a central role in orchestrating immune responses to diverse microbial pathogens [1]. Upon activation by antigens, naive CD4^+^ T cells differentiate into specialized effector T (Teff) cells (Th1, Th2, or Th17), which secrete different patterns of cytokines and perform different functions [1]. Th1 cells produce interferon-γ (IFN-γ) and tumor necrosis factor-α (TNF-α) and initiate cellular immune responses against intracellular pathogens. Th2 cells generate interleukin-4 (IL-4), IL-5 and IL-13 and promote humoral responses against extracellular parasites. Th17 cells make IL-17, IL-21 and IL-22 and confer immunity against extracellular bacteria and fungi. Moreover, activated CD4^+^ T cells also differentiate into regulatory T (Treg) cells, which express transforming growth factor-β (TGF-β), IL-10 and IL-35 and suppress the functions of Teff cells, thereby keeping immune responses in check.

Imbalance of Th cell differentiation and subsequent cytokine dysregulation is implicated in inflammatory and autoimmune diseases [2]. In particular, Th1 and Th17 cells and their signature cytokines IFN-γ and IL-17 have been shown to play a critical role in the development of autoimmune responses in many autoimmune diseases, including multiple sclerosis (MS) and rheumatoid arthritis [2-4]. In accordance with the significance of Th cell differentiation in animal physiology and pathology, the molecular mechanisms underlying this important process have been extensively investigated. In this regard, the signal transducers and activators of transcription (STAT) proteins are well known for their essential roles in transmitting cytokine-mediated signals and specifying Th cell differentiation [1,2]. In general, STAT4 is activated mainly by IL-12 and type I IFNs, and it functions predominantly in promoting Th1 cell differentiation. STAT6 is activated in response to IL-4 and functions as the molecular switch for initiation of the Th2 cell differentiation program. Soon after activation by IL-6, STAT3 triggers Th17 commitment. On the other hand, IL-2-activated STAT5 facilitates Treg cell differentiation. Similar to STAT proteins, the NF-κB transcription factors, particularly the prototypical member RelA (also known as p65), are also master regulators/activators of immune responses and inflammation in both healthy and disease [5,6]. The signaling pathways leading to activation of STAT and NF-κB proteins have been well demonstrated [7,8]. However, it still remains largely unknown how activated STAT and NF-κB are terminated for proper Th cell differentiation and immune responses and how STAT and NF-κB are deregulated in autoimmune diseases.

Previous studies show that PDLIM2, a ubiquitously expressed PDZ-LIM domain-containing protein with high expression in lymphoid tissues and cells including T lymphocytes, is required for the termination of STAT and NF-κB activation [9,10]. More recent studies suggest that PDLIM2 may function as a tumor suppressor [11-15]. Mechanistic studies indicate that PDLIM2 selectively promotes ubiquitination and proteasomal degradation of nuclear (activated) STAT4 and RelA proteins [9-12]. However, whether and how PDLIM2 is involved in Th cell differentiation remain unknown. In particular, mouse genetic studies reveal that PDLIM2 is not required for the development of immune cells and immune tissues/organs [9]. Additionally, it remains unknown whether PDLIM2 is involved in the pathogenesis of inflammatory and autoimmune diseases.

## Results and discussion

### PDLIM2 deficiency in CD4^+^ th cells enhances Th1 and Th17 cell differentiation but has no obvious effect on Th2 and Treg cell differentiation

**Figure 1:**
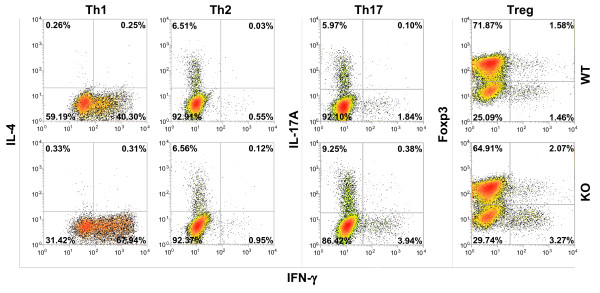
**Enhanced Th1 and Th17 differentiation of PDLIM2 deficient CD4**^**+**^**Th cells.** Naive CD4^+^ Th cells isolated from PDLIM2^+/+^ (WT) or PDLIM2^−/−^ (KO) mice were stimulated for 72 hours with anti-CD3/anti-CD28 under Th1, Th2, Th17 or Treg polarizing condition, followed by intracellular cytokine staining and flow cytometry. The data are representative of at least three independent experiments with similar results.

To test whether PDLIM2 is involved in Th cell differentiation, naive CD4^+^ Th cells were isolated from spleens of PDLIM2^−/−^ and PDLIM2^+/+^ mice and stimulated by anti-CD3/anti-CD28 under Th1, Th2, Th17 or Treg polarizing condition. Loss of PDLIM2 did not affect the differentiation of Th cells to Th2 or Treg, as evidenced by similar numbers of Th2 and Treg cells produced from naive PDLIM2^−/−^ and PDLIM2^+/+^ CD4^+^ Th cells (Figure 1). In contrast, much more Th1 and Th17 cells were generated from naive PDLIM2^−/−^ CD4^+^ Th cells compared to PDLIM2^+/+^ cells. These data suggest that PDLIM2 plays a specific role in restricting Th1 and Th17 cell differentiation.

### Mice deficient in PDLIM2 show increased susceptibility to EAE

**Figure 2:**
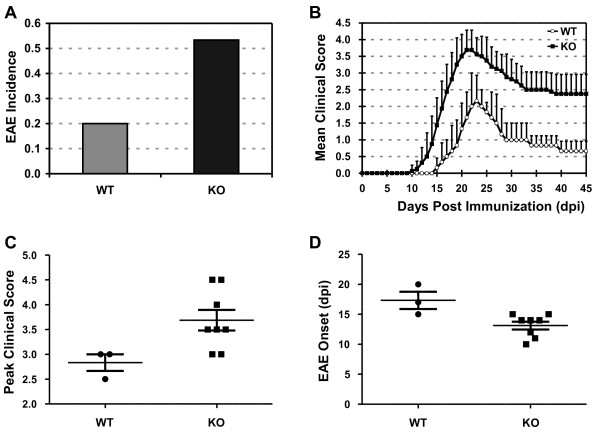
**Increased susceptibility to EAE in PDLIM2 deficient mice.A**) Incidence, **B**) disease progression, **C**) severity and **D**) onset of EAE in PDLIM2^+/+^ and PDLIM2^−/−^ mice (n = 15). Mice were immunized with PLP_180–199_ peptide and monitored daily for EAE disease symptoms. The p values between the PDLIM2^+/+^ (WT) and PDLIM2^−/−^ (KO) groups are at least smaller than 0.05 by two tailed *t*-test.

Given the causative role of Th1 and Th17 cells in autoimmune diseases such as MS [2-4], we proposed that through restriction of Th1 and Th17 cell differentiation, PDLIM2 is involved in autoimmune disease suppression. To test this hypothesis and to further characterize the *in vivo* role of PDLIM2 in regulating Th1 and Th17 cell differentiation, we examined the susceptibility of PDLIM2^−/−^ and PDLIM2^+/+^ mice to EAE, a well-defined model of MS [16]. In agreement with previous studies [17], 20% of PDLIM2^+/+^ mice developed acute EAE with a 2.8 mean peak clinical score and a mean disease onset of day 17.3 ± 2.5) of post-immunization with the encephalitogenic PLP_180-199_ epitope (Figure 2). Remarkably, over 50% of PDLIM2^−/−^ mice developed EAE with an earlier disease onset (13.1 ± 1.9 day of post-immunization) and a more severe (3.7 mean peak clinical score) and prolonged disease course. These data clearly indicate that PDLIM2 plays a critical role in suppressing EAE.

### PDLIM2 expression in CD4^+^ T cells is critical for EAE suppression

**Figure 3:**
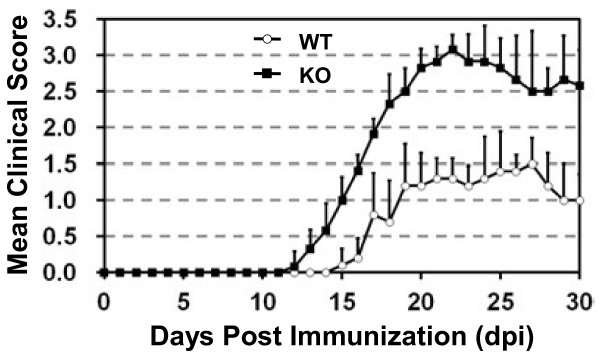
**Increased severity of adoptive transfer EAE in recipients of PDLIM2 deficient CD4**^**+**^**T cells.** CD4^+^ T cells were isolated from PDLIM2^+/+^ and PDLIM2^−/−^ mice immunized with PLP_180–199_ peptide and transferred i.v. into SCID recipients (n = 20). One day after the cell transfer, recipient mice also received an injection of pertussis. Mice were then monitored for the symptoms of EAE as described in Figure .2

To determine whether the effect of PDLIM2 deficiency on EAE is CD4^+^ T-cell specific, we performed adoptive CD4^+^ T-cell transfer studies using SCID mice as receipts, which lack CD4^+^ T cells. Although the disease severity in adoptive transfer recipients was less robust overall than that observed in immunized mice, the difference of EAE induction in the receipts of PDLIM2^+/+^ versus PDLIM2^−/−^ T cells was still significant and similar to that observed in PDLIM2^+/+^ and PDLIM2^−/−^ mice (Figure 3). These data suggest that the observed increase in EAE severity in PDLIM2^−/−^ mice is due to the deficiency of PDLIM2 in CD4^+^ T cells.

### PDLIM2 deficiency leads to increased STAT and NF-κB activation and augmented production of Th1 and Th17 cytokines

**Figure 4:**
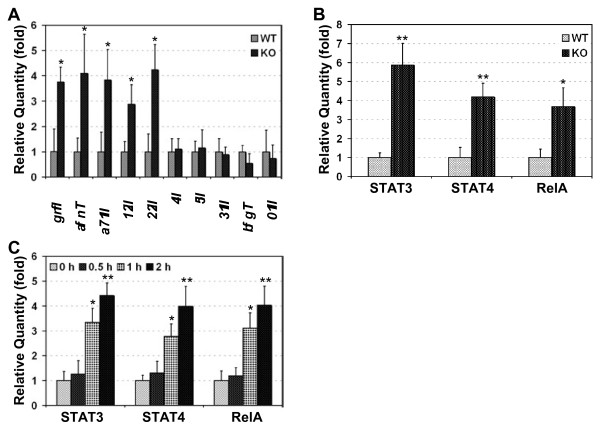
**Enhanced nuclear expression of STAT3/4 and RelA proteins and augmented production of Th1 and Th17 cytokines in PDLIM2 deficient Teff cells.** Splenic T cells from day 10 PLP_180–199_-immunized PDLIM2^+/+^ (WT) or PDLIM2^−/−^ (KO) mice were subjected to QRT-PCR to detect the relative expression levels of the indicated cytokines genes (**A**) or ELISA to detect the nuclear expression levels of STAT3, STAT4 and RelA (**B**). The expression levels of the indicated genes and proteins were represented as fold induction relative to their WT controls. **C**) Naive PDLIM2^−/−^ or PDLIM2^+/+^ CD4^+^ Th cells were stimulated for the indicated time points with anti-CD3/anti-CD28 under Th1 or Th17 polarizing condition, followed by ELISA to detect the nuclear expression levels of STAT3 (in response to Th17 stimulation), STAT4 and RelA (in response to Th1 stimulation). In A-C, n = 3, *, p < 0.03; **, p < 0.003 by two tailed *t*-test.

As EAE is mediated by Th1 and/or Th17 cells [3], we examined whether the exacerbated EAE in PDLIM2^−/−^ mice is associated with increased Th1 and Th17 cell differentiation in the mice. As expected, the expression levels of Th1 cytokines (IFN-γ and TNF-α) and Th17 cytokines (IL-17, IL-21 and IL-22) were significantly higher in PLP_180-199_-stimulated PDLIM2^−/−^ mice compared to the PDLIM2^+/+^ mice under the same treatment (Figure 4A). On the other hand, the expression levels of Th2 cytokines (IL-4, IL-5 and IL-13) and Treg cytokines (TGF-β and IL-10) were comparable in the PLP_180-199_-treated PDLIM2^+/+^ or PDLIM2^−/−^ mice. These data suggest that PDLIM2 suppresses EAE through limiting Th1 and Th17 cell differentiation.

**Figure 5:**
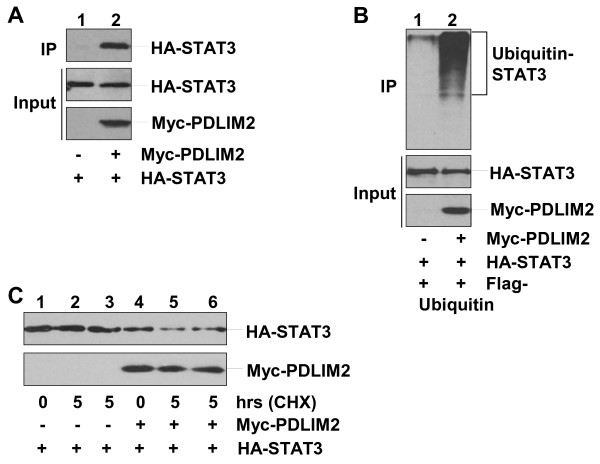
**Ubiquitination and proteasomal degradation of STAT3 by PDLIM2.A**) Physical interaction between PDLIM2 and STAT3. Nuclear extracts of 293 cells transfected with HA-STAT3 alone or together with Myc-PDLIM2 were subjected to immunoprecipitation (IP) using Myc antibody and immunoblotting (IB) using HA antibody. The expression levels of HA-STAT3 and Myc-PDLIM2 were examined by IB. **B**) Polyubiquitination of STAT3 by PDLIM2. 293 cells were transfected with HA-STAT3 plus Flag-ubiquitin in the presence or absence of Myc-PDLIM2, followed by nuclear fractionation. The nuclear extracts were subjected to IP using HA antibody and IB using Flag antibody. The expression levels of HA-STAT3 and Myc-PDLIM2 were examined by IB. **C**) Proteasomal degradation of STAT3 by PDLIM2. 293 cells transfected with HA-STAT3 alone or together with Myc-PDLIM2 were cycloheximide (CHX)-chased for the indicated time, followed by nuclear extractions and IB using HA or Myc antibody. In lanes 3 and 6, the cells were chased in the presence of 10 μM MG132.

To determine the molecular mechanisms by which PDLIM2 controls Th1 and Th17 cell differentiation for EAE suppression, we examined the expression levels of STAT4 and RelA proteins in the nucleus (activation marker) of CD4^+^ T cells isolated from PLP_180-199_-treated PDLIM2^+/+^ mice or PDLIM2^−/−^ mice. In this regard, it is known that PDLIM2 promotes proteasomal degradation of nuclear STAT4 and RelA proteins [9-12]. More importantly, STAT4 is a determinative factor of Th1 cell differentiation and also participates in Th17 cell differentiation [18,19]. On the other hand, RelA regulates transcriptional expression of numerous cytokines that are involved in Th1 and Th17 cell differentiation and EAE pathogenesis such as IFNs, TNF-α and IL-6 [6]. In fact, a recent study has already linked RelA to Th17 response [20]. Given the critical role of STAT3 in Th17 cell differentiation [21], we also included STAT3 in our studies. As shown in Figure 4B, significantly higher levels of STAT3, STAT4 and RelA proteins were detected in PLP_180-199_-treated T cells from PDLIM2^−/−^ mice as compared to those from PDLIM2^+/+^ mice. The increased nuclear expression/activation of STAT3, STAT4 and RelA should be the driving force but not the consequences of enhanced Th1 and Th17 cell differentiation nor the outcome of exacerbated EAE in PDLIM2^−/−^ mice, because an obvious increase in the nuclear expression of STAT3, STAT4 and RelA proteins was already detected within 30 minutes after cell stimulation (Figure 4C). Our biochemical studies indicated that similar to its role in the negative regulation of STAT4 and RelA (9–12), PDLIM2 bound to nuclear STAT3 for ubiquitination and proteasomal degradation (Figure 5). During the preparation of our manuscript, another group also showed that PDLIM2 targets STAT3 for degradation [22]. These data together suggest that PDLIM2 negatively regulates activation of STAT3/4 and RelA and therefore restricts Th1 and Th17 cell differentiation and prevents EAE development.

The STAT and NF-κB transcription factors play critical roles at multiple levels of the immune system in both health and disease, including the autoimmune inflammatory response [1-6]. The mechanisms of how STAT and NF-κB are activated to drive immune responses have been well defined [7,8]. However, how those key immune regulators are negatively regulated during Th cell differentiation and how they become constitutively and persistently activated in autoimmune diseases remain largely unknown. The data presented in this study demonstrate that PDLIM2 functions as an essential modulator of Th1 and Th17 cell differentiation but has no apparent effect on Th2 and Treg cell differentiation. Interestingly, the novel function of PDLIM2 in Th cell differentiation is most likely through restricting activation of STAT3/4 and RelA. These data identify STAT3 as a new target of PDLIM2 for ubiquitin-mediated proteasomal degradation and also suggest a new mechanism of RelA in immune responses involving regulation of Th1 and Th17 cell differentiation. These findings provide important insights into molecular mechanisms underlying immune responses and suggest PDLIM2 as a new therapeutic target for inflammatory and autoimmune diseases.

## Methods

### Mice

PDLIM2^−/−^ mice were backcrossed with BALB/c mice at least 10 generations for pure BALB/c background. PDLIM2^−/−^ BALB/c mice and control PDLIM2^+/+^ BALB/c mice were housed under specific pathogen-free conditions at the Hillman Cancer Center of the University of Pittsburgh Cancer Institute. Animal experiments were approved by the Institutional Animal Care and Use Committee (IACUC) of the University of Pittsburgh.

### Experimental autoimmune encephalitis (EAE) induction and clinical scoring

Six to eight-week-old female mice were immunized subcutaneously with PLP_180–199_ peptide (200 μg/mouse, Genemed Synthesis Inc.) emulsified in CFA containing *Mycobacterium tuberculosis* H37Ra (500 μg/mouse, BD Diagnostics). Mice also received 300 ng of pertussis toxin (List Biological Laboratories) intraperitoneally (i.p.) at the time of immunization and 48 hours later. Mice were monitored daily for clinical signs of paralysis and scored as follows: 0, no clinical signs; 1, limp tail; 2, weak/partially paralyzed hind legs; 3, limp tail and complete paralysis of hind legs; 4, complete hind and partial front leg paralysis; 5, complete paralysis or moribund state.

### Adoptive transfer of CD4^+^ T cells for induction of EAE

Lymph nodes and spleens were harvested from PDLIM2^+/+^ or PDLIM2^−/−^ mice immunized with PLP_180–199_, and lymph node cells and splenocytes were cultured *in vitro* with 1 μM PLP_180–199_ and IL-2 for 72 h. CD4^+^ T cells were then positively selected by MACS separation using magnetic CD4^+^ microbeads (Miltenyi Biotec, Auburn, CA) per manufacturer’s instructions. 5 x 10^6^ CD4^+^ T cells were adoptively transferred by intravenous (i.v.) injection into SCID recipients on day 0. On day 2, mice received an i.p. injection of pertussis toxin (250 ng), and mice were then monitored for symptoms of disease.

### CD4^+^ th cell purification and *in vitro* differentiation

Naive CD4^+^CD25^-^ T cells were first isolated from splenocytes using CD4^+^ T-cell Isolation Kit (Miltenyi Biotec.) and then sorted out by FACSAria (BD Biosciences). Purified naive CD4^+^CD25^-^ T cells were stimulated with plate-bound anti-CD3 and anti-CD28 (1 μg/ml) under Th1 (mIL-2 10 ng/ml, mIL-12 10 ng/ml), Th2 (IL-4 10 ng/ml, anti-IFNγ 10 μg/ml), Th17 (anti-IFNγ 10 μg/ml, anti-IL-4 10 μg/ml, hIL-6 10 ng/ml, hTGF-β 10 ng/ml) or Treg (hTGFβ, 10 ng/ml, anti-IL-4 10 μg/ml, anti-IFNγ 10 μg/ml) (BD Biosciences or eBioscience) polarizing condition. 72 hours after the initial stimulation, the cells were subjected to intracellular cytokine staining (ICS)/flow cytometry analysis and quantitative real-time RT-PCR (QRT-PCR) as described below.

### ICS and flow cytometry

T cells were stimulated for 5 hours with PMA (50 ng/ml) and ionomycin (500 ng/ml) in the presence of intracellular transport inhibitor monesin (10 μg/ml; Sigma), followed by fixation with paraformaldehyde (2%) and permeablization with saponin (0.5%). Cells were then treated with anti-IFN-γ-FITC (XMG1.2), anti-IL-4-PE (11B11), anti-IL-17-PE (TC11-18 H10), and anti-Foxp3–FITC (FJK-16 s) (BD Biosciences or eBioscience). Data were acquired using FACSCalibur (BD Biosciences) and analyzed using CellQuest software (Becton Dickinson) as described previously [23].

### QRT-PCR

Total RNA was prepared with TRIZOL reagent and cDNA was generated with SuperScript II reverse transcriptase (Invitrogen), followed by real-time PCR assays using Fast start SYBR Green reagents (Roche) as described [24,25]. The gene-specific primer pairs were: IFN-γ, 5’-TTCTTCAGCAACAGCAAGGCGAA-3’ and 5’-TGAATGCTTGGCGCTGGACCTG-3’; TNF-α, 5’-GATGAGAAGTTCCCAAATGGC-3’ and 5’-ACTTGGTGGTTTGCTACGACG-3’; TGF-β, 5’-TGACGTCACTGGAGTTGTACGG-3’ and 5’-GGTTCATGTCATGGATGGTGC-3’; IL-4, 5’-AGGGACGCCATGCACGGAGAT-3’ and 5’-GCGAAGCACCTTGGAAGCCCTAC-3’; IL-5, 5’-AGCACAGTGGTGAAAGAGACCTT-3’ and 5’-TCCAATGCATAGCTGGTGATTT-3’; IL-10, 5’-AGCTGAAGACCCTCAGGATGCG-3’ and 5’- TCATTCATGGCCTTGTAGACACCTTG-3’; IL-13, 5’-GGCTCTTGCTTGCCTTGGTG-3’ and 5’-TCCATACCATGCTGCCGTTG-3’; IL-17, 5’-CTCAGACTACCTCAACCGTTC-3’ and 5’-TGAGCTTCCCAGATCACAGAG-3’; IL-21, 5’-ATCCTGAACTTCTATCAGCTCCAC-3’ and 5’-GCATTTAGCTATGTGCTTCTGTTTC-3’; IL-22, 5’-TCCGAGGAGTCAGTGCTAAA-3’ and 5’-AGAACGTCTTCCAGGGTGAA-3’; β-actin, 5′-ACCCGCGAGCACAGCTTCTTTG-3’ and 5’-CTTTGCACATGCCGGAGCCGTTG-3’. Expression levels of each gene were normalized to that of β-actin.

### Enzyme-linked immunosorbent assay (ELISA)

Cell nuclear fractions were prepared and added to 96-well plate precoated with anti-RelA, anti-STAT3 or anti-STAT4. After overnight incubation at 4 °C, plates were washed extensively with PBS containing 0.1% Tween 20 (PBST), and horseradish peroxidase-conjugated secondary antibodies were added and incubated for 1 hour at room temperature. After extensive wash with PBST, a colorimetric substrate 2’2-azinobis(3-ethylenzthiazoline-6-sulfonic acid) (ABTS) was added and incubated for 15 minutes. The reaction was stopped by addition of 100 μL 1% sodium dodecyl sulfate (SDS). The optical density at 405 nm (OD405) was measured with an automated plate spectrophotometer (Thermo Lab Systems).

### Immunoblotting (IB) and immunoprecipitation (IP) assays

Nuclear extracts were subjected to SDS-PAGE and IB, or IP using the indicated antibodies before SDS-PAGE and IB as described before [26,27].

### *In vivo* ubiquitin conjugation assay

Cytoplasmic and nuclear extracts were prepared from HTLV-I-transformed T cells or 293 cells transfected with HA-STAT3 together with Flag-tagged ubiquitin in the presence or absence of Myc-PDLIM2, immediately followed by IP using anti-HA. The ubiquitin-conjugated STAT3 pulled down by IP was detected by IB using anti-Flag [28].

### Protein stability assay

Cells were treated with 10 μM CHX, followed by chase of the indicated time period in the presence or absence of MG132, and IB to detect the indicated proteins [29].

### Statistical analysis

Data were reported as mean ± standard deviation (SD). The Student’s *t* test (two tailed) was used to assess significance of differences between two groups, and p values ≤ 0.05 and 0.01 were considered statistically significant and highly statistically significant, respectively.

## Abbreviations

ABTS, 2’2-azinobis(3-ethylenzthiazoline-6-sulfonic acid); EAE, Experimental autoimmune encephalitis; ELISA, Enzyme-linked immunosorbent assay; IB, Immunoblotting (IB); ICS, Intracellular cytokine staining; IFN-γ, Interferon-γ; IL, Interleukin; IP, Immunoprecipitation; (i.p.), Intraperitoneal; (i.v.), Intravenous; MS, Multiple sclerosis; QRT-PCR, Quantitative reverse transcription- polymerase chain reaction; PDLIM2, PDZ-LIM domain-containing protein 2; STAT, Signal transducers and activators of transcription; SDS, Sodium dodecyl sulfate; TGF-β, Transforming growth factor-β; Th, T helper; Teff, Effector T; TNF-α, Tumor necrosis factor-α.

## Competing interest

The authors declare that they have no competing interests.

## Authors’ contributions

ZQ, JF, HM, JZ and MJ performed experiments; MM analyzed data and criticized the paper; MG contributed vital new reagents and criticized the paper; ZQ and GX designed the research, analyzed data and wrote the paper. All authors read and approved the final manuscript.
